# Microstructure Investigation of WC-Based Coatings Prepared by HVOF onto AZ31 Substrate

**DOI:** 10.3390/ma15010040

**Published:** 2021-12-22

**Authors:** Ewa Jonda, Leszek Łatka, Anna Tomiczek, Marcin Godzierz, Wojciech Pakieła, Paweł Nuckowski

**Affiliations:** 1 Department of Engineering Materials and Biomaterials, Silesian University of Technology, 18a Konarskiego Str., 44100 Gliwice, Poland; ewa.jonda@polsl.pl (E.J.); wojciech.pakiela@polsl.pl (W.P.); 2 Department of Metal Forming, Welding and Metrology, Faculty of Mechanical Engineering, Wroclaw University of Science and Technology, 5 Łukasiewicza Str., 50371 Wroclaw, Poland; 3 Scientific and Didactic Laboratory of Nanotechnology and Material Technologies, Faculty of Mechanical Engineering, Silesian University of Technology, 7a Towarowa Str., 44100 Gliwice, Poland; anna.tomiczek@polsl.pl; 4 Centre of Polymer and Carbon Materials Polish Academy of Sciences, 34 M. Curie-Skłodowskiej Str., 41819 Zabrze, Poland; 5 Materials Research Laboratory, Faculty of Mechanical Engineering, Silesian University of Technology, 18a Konarskiego Str., 44100 Gliwice, Poland; pawel.nuckowski@polsl.pl

**Keywords:** High Velocity Oxy Fuel, AZ31 magnesium alloy, microstructure, X-ray diffraction, residual stress analysis—sin^2^ψ method

## Abstract

In this paper, three commercial cermet powders, WC-Co-Cr, WC-Co and WC-Cr_3_C_2_-Ni, were sprayed by the High Velocity Oxy Fuel (HVOF) method onto magnesium alloy AZ31 substrate. The coatings were investigated in terms of their microstructure, phase analysis and residual stress. The manufactured coatings were analyzed extensively using optical microscopy (OM), X-ray diffraction (XRD), scanning (SEM) and transmission electron microscopy (TEM). Based on microstructure studies, it was noted that the coatings show satisfactory homogeneity. XRD analysis shows that in WC-Co, WC-Co-Cr and WC-Cr_3_C_2_-Ni coatings, main peaks are related to WC. Weaker peaks such as W_2_C, Co_0.9_W_0.1_, Co and W for WC-Co and W_2_C, Cr_3_C_2_ and Cr_7_C_3_ for WC-Cr_3_C_2_-Ni also occur. In all cermet coatings, linear stress showed compressive nature. In WC-Co and WC-Cr_3_C_2_-Ni, residual stress had a similar value, while in WC-Co-Cr, linear stress was lower. It was also proved that spraying onto magnesium substrate causes shear stress in the WC phase, most likely due to the low elastic modulus of magnesium alloy substrate.

## 1. Introduction

The elements of machines and equipment operated in the conditions of abrasive, erosion or corrosion wear and tear are exposed to damage. New element production costs significantly exceed its recovery enabling the restoration of its usable values and increased durability. One of the numerous methods allowing for a combination of the beneficial properties of the core with resistance to abrasive wear, heat resistance and increased hardness is the deposition of coating [1,2]. This enables lifetime increase and improvement of reliability and operating durability of the machinery elements. One of the most frequently applied technologies of protective coating deposition is thermal spraying [3,4,5]. Generally, it produces metallic, carbide, ceramic and composite coatings of any chemical and phase composition on an appropriately prepared base [6,7]. Among the most commonly used methods mentioned above is the HVOF (High Velocity Oxy Fuel) spraying method, which enables the production of dense coatings with compact structure and high adhesion to the substrate. In the HVOF method, the flammable gas (or liquid fuel) is fed to the combustion chamber together with the oxygen, and the stream of gases produced during combustion is formed in the nozzle. The HVOF gun works continuously while the liquid fuel is sprayed. Among the most frequently used flammable gases, ethylene, propylene and acetylene should be mentioned, while kerosene is a popular liquid fuel. In the stream of argon or nitrogen, the feedstock material is fed (mainly in the form of powder) axially or radially along the gun axis [1,8]. The most significant applications of the HVOF method include spraying cermet coatings based on tungsten carbide (WC), where, due to the low temperature obtained by the particles, carbide transformation takes place to a lesser extent. The cermet materials are a combination of metallic and metal materials in which the substrate is usually formed from Co, Ni, Al, Ti, Mo or their alloys. Because the tungsten carbide (WC) could be well wetted, without limitation, by cobalt (Co), nickel (Ni), iron (Fe) and cobalt–chromium (CoCr), the cermet materials based on WC are some of the most frequently used cermet materials [9,10,11,12]. The advantage of these materials is their high resistance to abrasive, erosion and cavitation wear and the ceramic coatings produced from them are characterized, without limitation, with higher hardness, low thermal conductivity coefficient, high corrosion and oxidation resistance and high resistance to abrasion and erosion [13,14,15].

In the literature, there is a small gap concerning the deposition of hard and wear-resistance coatings on soft and low melting temperature substrate. It is much more important when combined with mass reduction, e.g., in the automotive and aviation industry. This study’s novelty is the purpose of using a magnesium alloy as a substrate. This group of materials has not yet been investigated in depth. Magnesium alloys are lighter than aluminum ones, which is a significant advantage. However, the main disadvantages are poor mechanical properties and resistance against wear, erosion, corrosion, etc. In recent years, some investigations have mainly concentrated on HVOF coating materials, such as amorphous Fe-based, stainless steel and hydroxyapatite [16,17,18]. Only a few articles are dedicated to cermet HVOF coating on magnesium alloy substrate [16,17]. Cermet coatings produced by HVOF could substantially improve these properties on the top surface. Moreover, because of relatively soft flame and average temperatures (ensured by an appropriate selection of process parameters), HVOF spraying allows manufacturing such coatings onto magnesium substrate without damaging it [19,20].

This study’s main aim was to investigate and compare the microstructure and residual stress in the cermet coatings manufactured by the HVOF method on the AZ31 magnesium alloy substrate. Such a solution could make it possible to use it in aircraft structures.

## 2. Materials and Methods

### 2.1. Powders

In this study, three commercially available powders were used as feedstock material. They are labelled as follows:

- P1-WC-Co-Cr (86-10-4, Höganäs, Amperit 558.074);
- P2-WC-Co (88-12, Höganäs, Amperit 518.074); and
- P3-WC-Cr_3_C_2_-Ni (73-20-7, Woka 3702-1).

Chemical compositions have been given in wt %. For all powders, delivery conditions were agglomerated and sintered. Moreover, the particle size range was −45 + 15 µm for each one. The main diameter d_50_ was around 30 µm for all powders.

### 2.2. Deposition Process

**Figure 1:**
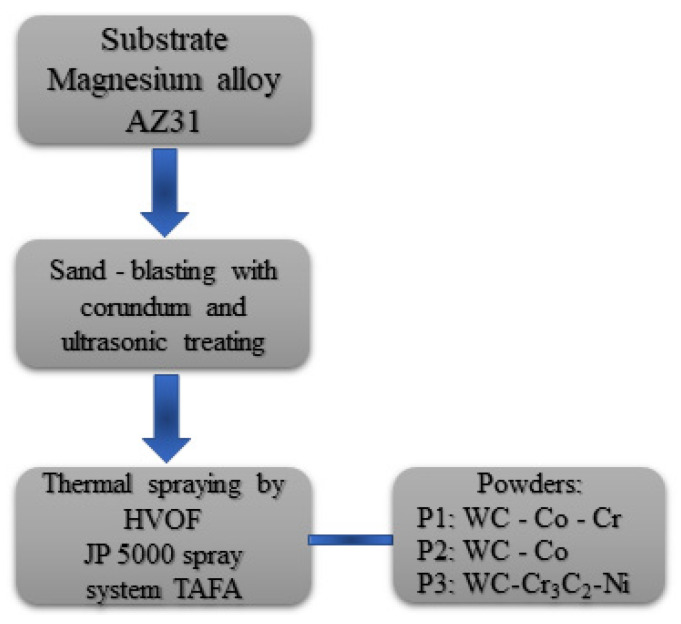
The schematic diagram of the HVOF coating manufacturing process.

The magnesium alloy AZ31 with 5 mm thickness was used as a substrate. Before the spraying, the surfaces of the samples were sand-blasted with corundum and ultrasonic treated. The JP 5000 spray system TAFA (Indianapolis, IN, USA) by RESURS (Warszawa, Poland) was used to manufacture the coatings. Kerosene and oxygen were used as the fuel media, whereas nitrogen was used as the carrier gas. The schematic diagram of the HVOF coating process is presented in Figure 1, and the spraying parameters are listed in Table 1. The coatings manufactured from P1, P2 and P3 powders are labelled in the text as C1, C2 and C3, respectively.

### 2.3. Coatings’ Characterization

Microscopic investigations and fracture morphology were carried out by scanning electron microscope (Supra 35, Zeiss, Oberkochen, Germany) with secondary electron and backscattered detectors. The chemical composition was analyzed by EDS (energy dispersive X-ray spectroscopy) (Supra 35, Zeiss, Oberkochen, Germany). The area EDS measurements were randomly distributed in the coating. It was carried out in one sample in 10 areas, and three coatings were tested. TEM investigations were undertaken with a field emission transmission electron microscope (S/TEM Titan 80-300 from FEI, Hillsboro, OR, USA) with a super twin-lens operated at 300 kV and equipped with an annular dark-field detector. A focused ion beam method (FIB) prepared thin foils for TEM analysis. The lamella extraction was performed on the SEM/Ga-FIB FEI Helios NanoLab 600i (FEI, Brno, Czech Republic) device, while the thinning and removal of the amorphous layer were performed on SEM/Xe-PFIB FEI Helios G4 PFIB CXe (FEI, Brno, Czech Republic). The coatings’ cross-sections were observed by a Keyence VHX6000 (Keyence International, Mechelen, Belgium) microscope. Based on these images, at 2000× magnification, the porosity of sprayed coatings was estimated according to ASTM E2109-01 standards. Image J open-source software (1.50i version) was used to calculate porosity. At the same magnification, 10 measurements carried out at random locations along the coatings cross-sections were taken into account to calculate the average thickness value and standard deviation.

Microhardness of manufactured coatings were estimated with Vickers indenter under the load of 2.94 N (HV0.3) using the HV1000 hardness tester (Sinowon Innovation Metrology), according to the ISO 4516 standard. Ten imprints at the cross-sections of each coating were made to calculate the average value and standard deviation.

XRD studies were performed using the D8 Advance diffractometer (Bruker, Karlsruhe, Germany) with a Cu-Kα cathode (λ = 1.54 Å) operating at 40 kV voltage and 40 mA current. The scan rate was 0.60°/min with a scanning step of 0.02° in the range of 20° to 120° 2Θ. Identification of fitted phases was performed using the DIFFRAC.EVA program using the ICDD PDF#2 database, while the exact lattice parameters of the fitted phase were calculated using Rietveld refinement in the TOPAS 6 program, based on the Williamson–Hall theory [21,22,23]. The pseudo-Voigt function described diffraction line profiles at the Rietveld refinement. The Rwp (weighted-pattern factor) and S (goodness-of-fit) parameters were used as numerical criteria of the quality of the fit of calculated to experimental diffraction data.

Residual stress analyses (RSA) were performed using the iso-inclination mode of the D8 Advance diffractometer (Bruker, Karlsruhe, Germany) with the use of the (211) peak of the WC phase, according to EN-15305 standards. RSA measurements were performed at six different φ angles (0°, 45°, 90°, 135°, 180°, 225°) to obtain a reliable stress mode [24,25]. Results were evaluated using the DIFFRAC.LEPTOS program, and all peaks were fitted using standard fit, while the applied stress mode was established as biaxial [24,25] with consideration of shear stress contribution, due to low hardness and elastic modulus of substrate material, which is mainly omitted in literature. The following material parameters were used for residual stress analysis: Young’s modulus 600 GPa and Poisson ration 0.20, which gives S_1_ = −3.333 10^−7^ MPa and 1/2S_2_ = 2.000 10^−6^ MPa^−1^ and are in agreement with literature data [25]. The 45 MPa limit was used as a stress-free WC material, while a 22.5 MPa limit was used for shear stress contribution.

## 3. Results and Discussion

### 3.1. Feedstocks

**Figure 2:**
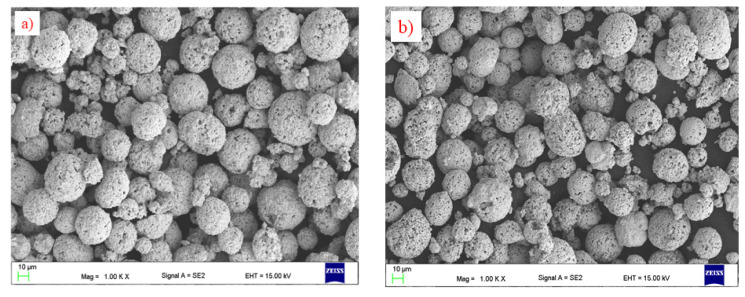

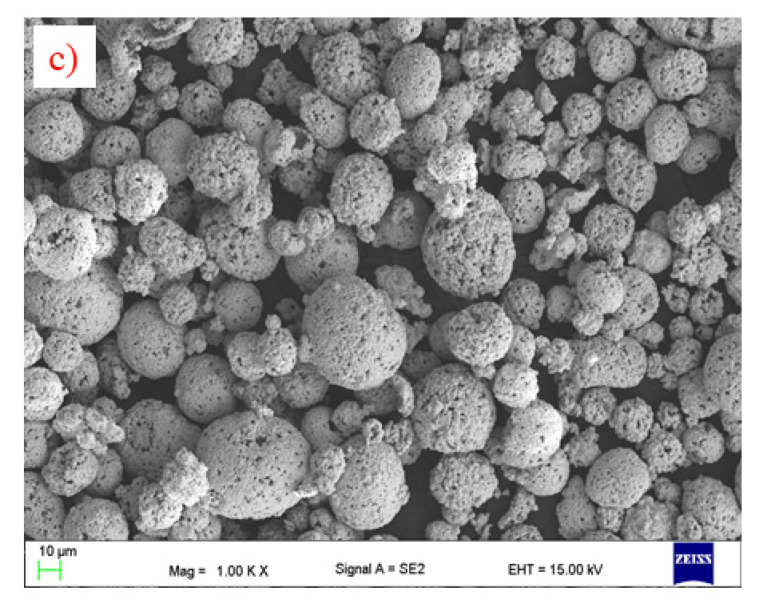
Morphology of: (**a**) P1, (**b**) P2, and (**c**) P3 powder (SEM).

The morphology of feedstock powders is given in Figure 2. All powders have similar particles size and spherical shapes. This is important from a technological point of view because it provides suitable flowability of the powder particles during spraying.

### 3.2. Microstructure of the Coatings

**Figure 3:**
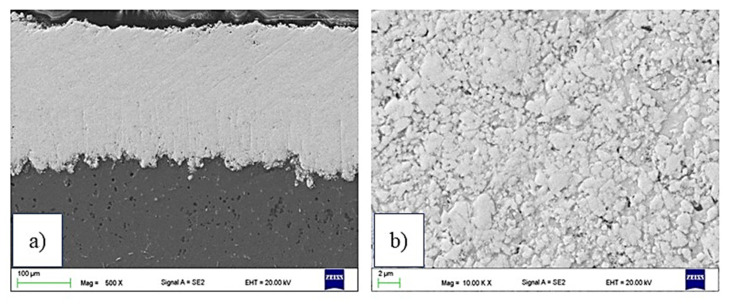

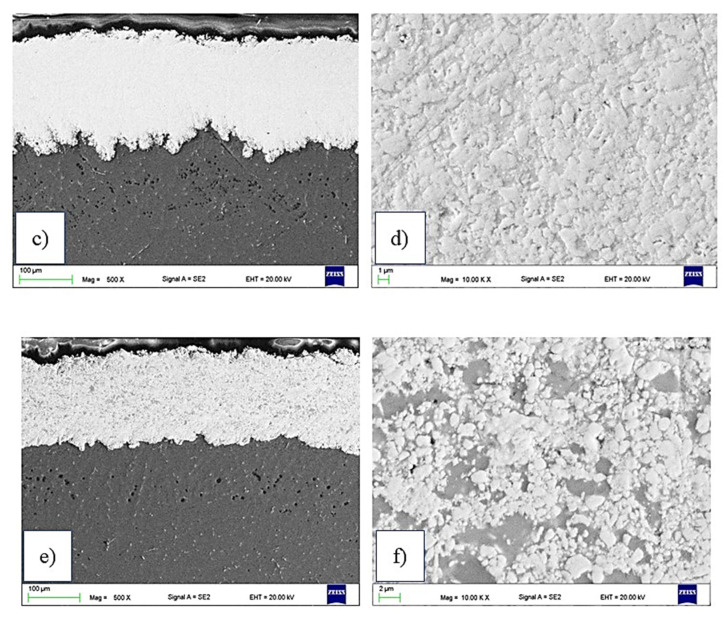
SEM images of cross-sections of HVOF-sprayed coatings: (**a**,**b**) C1, (**c**,**d**) C2, (**e**,**f**) C3 ((**a**,**c**,**e**)—mag. 500×; (**b**,**d**,**f**)—mag. 10,000×).

The detailed examination at high magnification (Figure 3b,d,f) revealed a dense structure with fine pores (much lower than 1 µm) and a typical low porosity level. This dense structure is due to the inherent characteristic of the HVOF process (mainly the high kinetic energy of the particles). In Figure 3b,d, the hard particles are homogeneously distributed in the cobalt matrix, whereas for C3 (Figure 3f), there are some areas of nickel matrix islands without hard particles. This is similar to the phenomenon reported by [26,27,28].

The examination of low-magnification polished cross-sections (Figure 3a,c,e) showed a relatively smooth, dense and homogeneous structure of HVOF-sprayed coatings. The microstructure is typical for thermal spraying coatings. The interface between cermet coating and AZ31 substrate was clear in all samples, and no evidence of delamination was observed.

The image analysis results in coatings’ porosity determination are collected in Table 2, and the results are quite similar. The lowest porosity value for the C3 sample could be related to lower hardness and better porosity filling by nickel than cobalt. In their work, Yao et al. [13] reported that the coating porosity is related to the powder composition and oxygen flow rate, and decreased with the oxygen flow increase. In Table 2, the coatings’ thickness values and microhardness (HV0.3) are presented.

**Figure 4:**
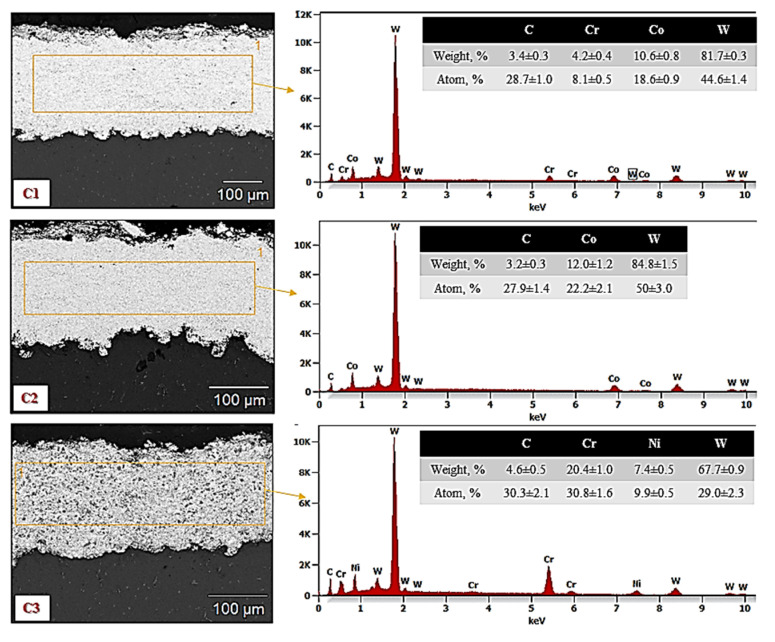
The chemical composition of the HVOF-sprayed coatings.

**Figure 5:**
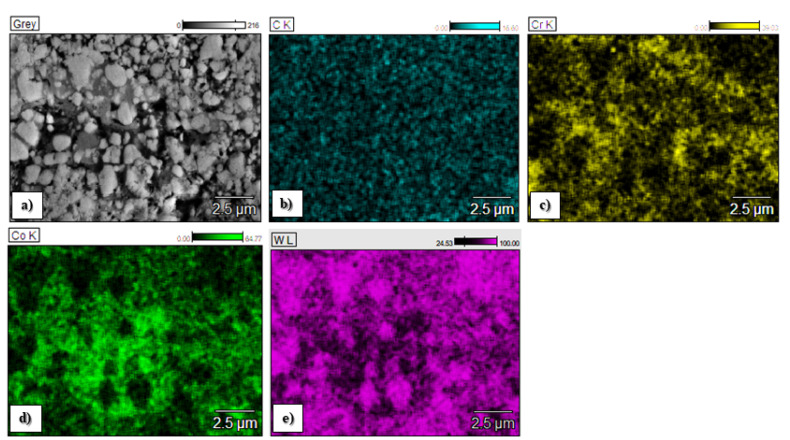
Elemental distribution maps of spraying elements in the analyzed area of the C1 coating obtained during thermal spraying: (**a**)—central part of the layer, (**b**)—map of the carbon, (**c**)—map of the chromium, (**d**)—map of the cobalt, (**e**)—map of the tungsten.

**Figure 6:**
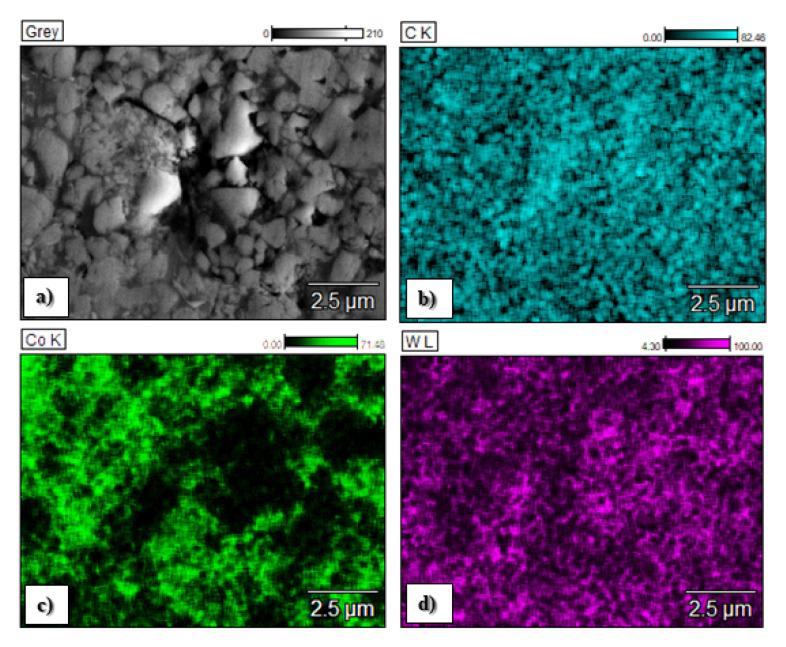
Elemental distribution maps of spraying elements in the analyzed area of the C2 coating obtained during thermal spraying: (**a**)—central part of the layer, (**b**)—map of the carbon, (**c**)—map of the cobalt, (**d**)—map of the tungsten.

**Figure 7:**
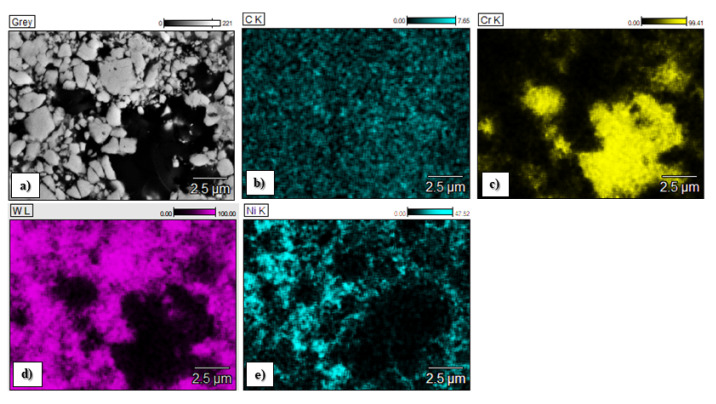
Elemental distribution maps of spraying elements in the analyzed area of the C3 coating obtained during thermal spraying: (**a**)—central part of the layer, (**b**)—map of the carbon, (**c**)—map of the chromium, (**d**)—map of the tungsten, (**e**)—map of the nickel.

The chemical composition of the C1, C2 and C3 sprayed coatings is presented in Figure 4, and the chemical element distributions in the micro areas are shown in Figure 5, Figure 6 and Figure 7.

The map analysis revealed the areas with a higher concentration of individual chemical elements in the analyzed coatings. In the case of the sample C1, the highest concentration of tungsten (light area in Figure 5a and purple in Figure 5e) and Cr (black area in Figure 5a and yellow area in Figure 5c), as well as Co (a gray area in Figure 5a and yellow Figure 5d), was observed. The increased share of these elements corresponds to tungsten carbide and a metallic CoCr matrix, respectively. Analysis of the distribution of elements in the area of the C2 coating showed an even distribution of tungsten carbide (light area in Figure 6a and purple area in Figure 6d) in the Co matrix (a gray area in Figure 6a and green in Figure 6c). In sample C3, areas with a large mass fraction of chromium (black area in Figure 7a and yellow in Figure 7c) and tungsten (bright area in Figure 7a and purple in Figure 7d), as well as a nickel (a gray area in Figure 7a and blue in Figure 7e) were observed, which correspond to carbides and a metallic Ni matrix used during the process.

The microhardness of the coatings depends on several factors, including porosity, carbide particle size and degree of decarburization. Process parameters (among others, spray distance) determines the temperature of the particles during spraying, which has a significant effect on hardness value. The coatings’ hardness increases with increasing particle temperature. It could be explained that decarburization and dissolution of W, Cr and C in the metal matrix (CoCr) take place at a higher temperature. Consequently, this leads to the hardness increasing. In general, the matrix hardness is higher and also W_2_C hard carbides are formed during spraying, which results in coatings’ hardness increasing. A similar value of microhardness and porosity for the C3 sample was observed in other investigations [29] and C1 and C2 samples [30]. Yuan et al. reported that the physical features such as morphology and density of the WC-Co powders play a very important role in determining the microhardness of the coatings by affecting the coating porosity and extent of decarburization [31].

**Figure 8:**
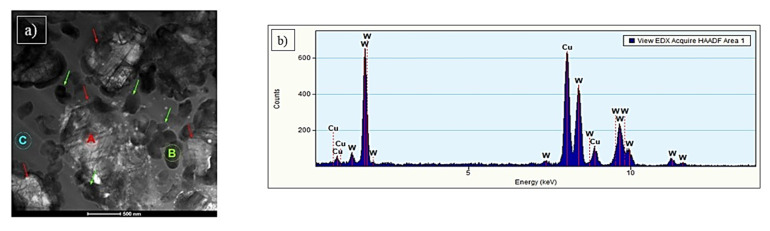
STEM micrograph in BF of sample C1 (**a**); the results of chemical analysis marked with letter A (**b**).

Results of TEM analysis are divided into three parts, according to the type of coating material. The C1 sample analysis revealed that coating contains a matrix and two-particle types (Figure 8). The particles marked with red arrows and named with the letter A (Figure 8a) are larger (1–2 μm) than others and irregular shapes. The analysis of the chemical composition (Figure 8b) confirmed the presence of tungsten (100 at. %).

**Figure 9:**
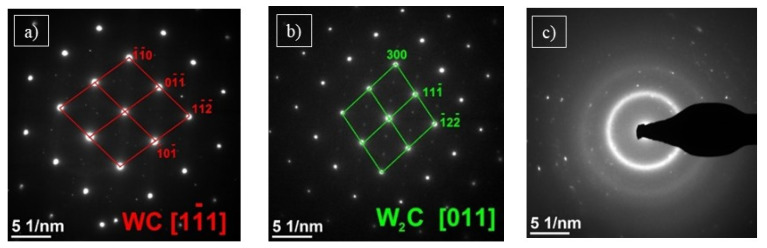
SAED diffraction analysis of sample C1; for the area marked on Figure 8a with letter A: WC [111] (**a**); with letter B: W2C [11] (**b**); with letter C as the matrix (**c**).

The spectrum of the energy-dispersive X-ray spectroscopy (EDS) also shows the signal from Cu, which was omitted in the analysis. It could results e.g., from holder and pole pieces. EDS technique has a limitation in the study of light elements (Z < 11). Based on the obtained spectrum, their presence (especially carbon) in the tested material cannot be excluded. Electron diffraction SAED (Figure 9a) identified the particles as WC, the hexagonal phase and the P-6m2 space group [32].

The second type of precipitation, marked with green arrows, has a more regular and spherical shape. It is occurring in the matrix or around the WC shown earlier. Diffraction investigation showed the W_2_C phase (Figure 9b), where Co and Cr replace some W atoms by the structure. The W_2_C phase belongs to the hexagonal system, space group P-3m1 [33]. Process parameters significantly influence the microstructure of the coating. Especially important is the point when high temperature affects particles of feedstock material. The 2000–3000 K W_2_C phase is more stable than the WC one in the temperature range.

Moreover, the higher temperature of the particles causes decarburization of the WC phase and leads to W_2_C precipitation, which is a harder and more brittle phase. Myalska et al., in their work, provided a detailed explanation of this phenomenon [34]. It was confirmed by SAED electron diffraction that the matrix is amorphous (Figure 9c).

**Figure 10:**
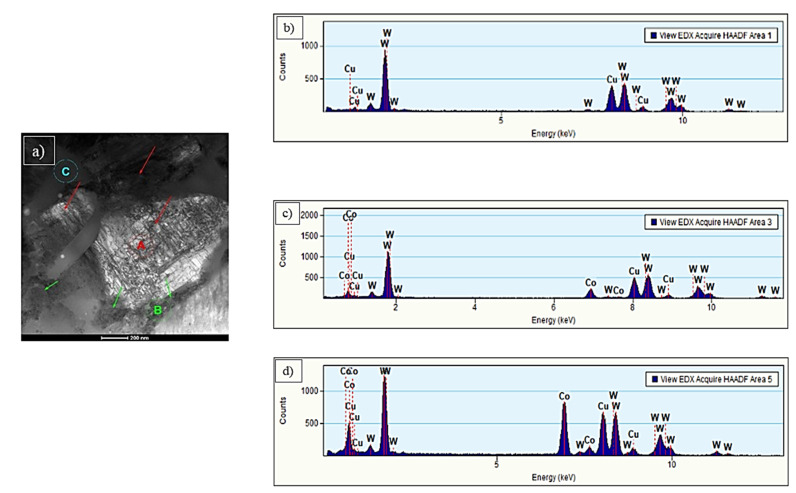
STEM micrograph in BF of sample C2 (**a**); (**b**) the results of chemical analysis for precipitation marked with letter A and (**c**) with letter B, (**d**) the results of chemical analysis for matrix marked with letter C.

**Figure 11:**
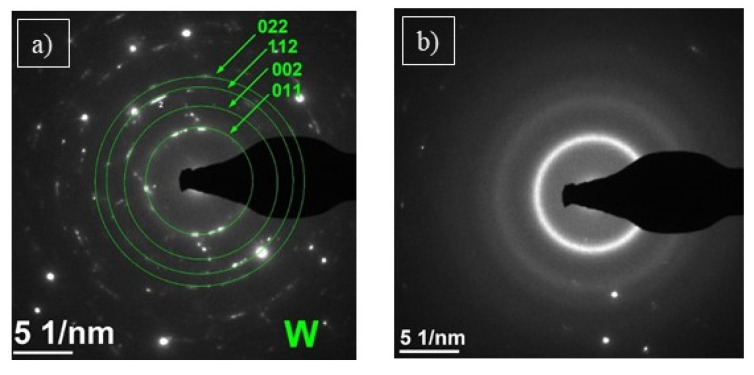
The SAED electron diffraction of sample C2, performed for the area marked as B (**a**) and amorphous matrix (**b**).

In the C2 sample, the precipitates marked with the letter B (Figure 10a) occur around larger WC carbides. They are irregular in shape and composed of many smaller grains. EDS analysis (Figure 10b) showed a high proportion of W (72 at. %) and Co (28 at. %). The SAED electron diffraction, performed for the area marked as B, confirmed the polycrystalline structure of the precipitation (Figure 11a). SAED diffraction identified the cubic tungsten with space group Im-3m [35]. The matrix has an amorphous structure, which may result from the high cooling rate of the particles while striking the substrate surface. It was confirmed by SAED electron diffraction (Figure 11b). The analysis of the chemical composition of the matrix (Figure 10c) confirmed the content of Co (58 at. %) and W (42 at. %). A similar morphology has been observed by other researchers [32,36,37].

**Figure 12:**
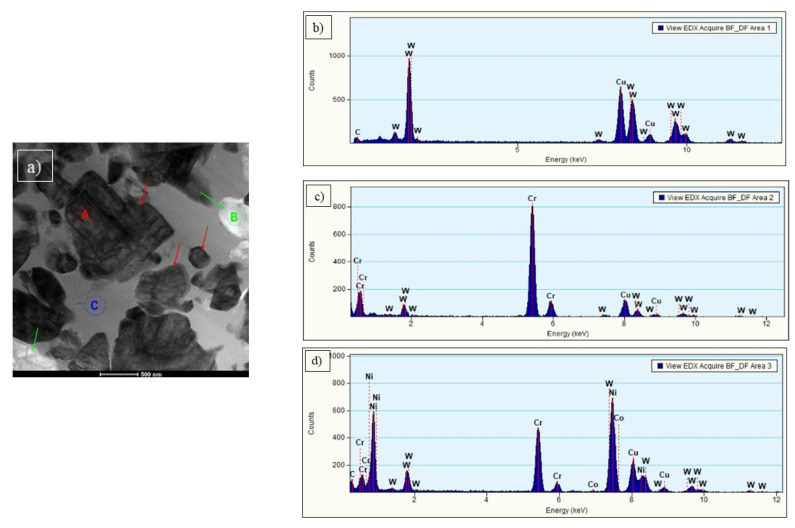
STEM micrograph in BF of sample C3 (**a**); the results of the chemical analysis performed for the area marked as A (**b**) and B (**c**) and C (**d**).

**Figure 13:**
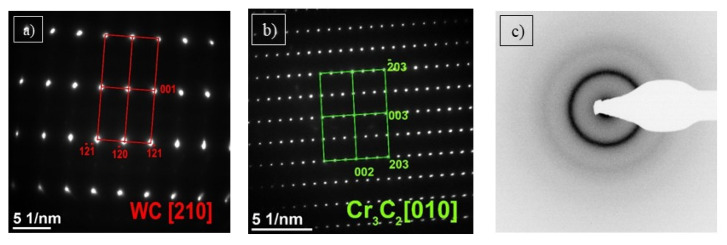
SAED diffraction analysis of sample C3; for the area marked on Figure 12a with letter A: WC with direction [210] (**a**); with the letter B: Cr_3_C_2_ with direction [10] (**b**); with letter C: amorphous matrix (**c**).

STEM analysis of C3 coating showed a matrix and two types of precipitations (Figure 12a). The ones marked by red arrows were identified as WC. They are characterized by irregular shapes and varied sizes (from 200 nm up to 1 µm). Chemical composition analysis (Figure 12b) confirmed the presence of W (100 at. %) inside these carbides. SAED pattern of WC phase and its solution with direction [210] is presented in Figure 13a. Separations marked by green arrows (Figure 12a) were identified as chromium carbide Cr_3_C_2_. They are rounded with size c.a. several hundred nanometers. Chemical composition analysis (Figure 12c) confirmed the presence of chromium (94 at. %) and tungsten (6 at. %). The SAED pattern of Cr_3_C_2_ is presented in Figure 13b. It is an orthorhombic space group Pnma [38]. SAED electron diffraction confirmed an amorphous structure of the matrix (Figure 13c). It could also be confirmed by uniform contrast in STEM bright-field images (Figure 12a).

### 3.3. Phase Composition

**Figure 14:**
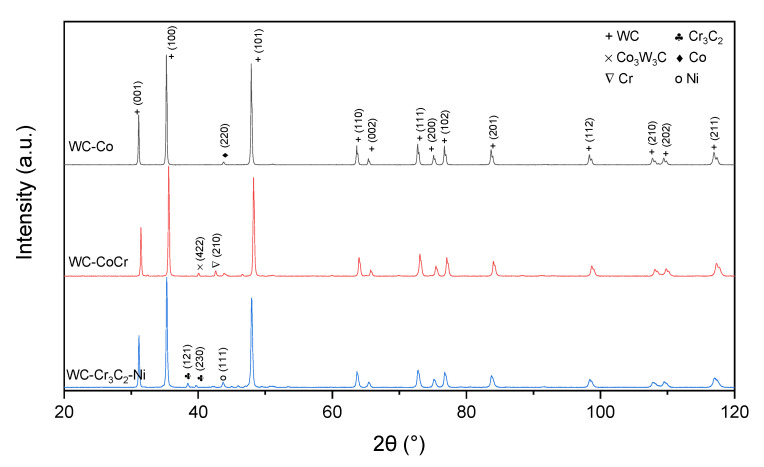
XRD patterns of feedstock powders.

Phase compositions of feedstock powders in the delivery conditions are shown in Figure 14. As expected, mainly the WC phase was detected. This phase composition was confirmed by other authors working with similar powders [34,36,39].

**Figure 15:**
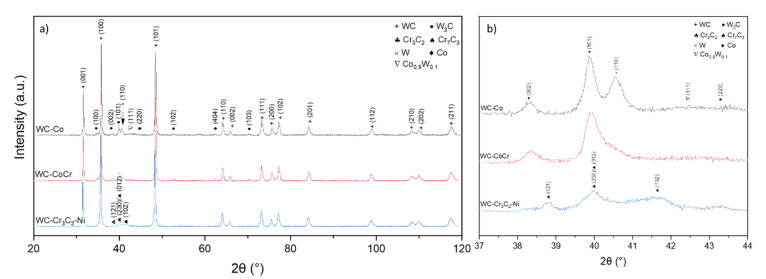
Typical XRD patterns obtained from cermet coatings sprayed onto magnesium substrate: (**a**) whole pattern, (**b**) magnification of chromium carbides region.

HVOF spraying resulted in changes in the coatings’ phase composition. The phase composition of cermet coatings consists of hexagonal WC (PDF#00-061-0244), hexagonal W_2_C carbide (PDF#00-035-0776), hexagonal Co (PDF#03-065-9722) and a cubic solid solution of W in Co with composition Co_0.9_W_0.1_ (PDF#03-065-9928). Additionally, in the C3 coating, the Cr_3_C_2_ (PDF#00-035-0804) and Cr_7_C_3_ (PDF#00-036-1482) carbides have been identified. Moreover, crystallites were detected in the C2 coating in the presence of cubic W (PDF#00-001-1204), which is in agreement with literature data [40]. It should be noted that no peaks coming either from the WC_1-x_ phase or from the Co_3_W_3_C or Co_6_W_6_C phases were found in the coatings, but those phases were identified in other papers and feedstock powders (Figure 15) [24,41,42,43]. During the deposition process of cermet coating, high temperature and oxygen lead to the decarburization process of carbides; thus, the formation of new carbides was detected instead of metal oxidation [24,41,42,43,44].

### 3.4. Residual Stress Analysis

The presence of residual stress in material might implicate unwanted effects during the exploitation of elements, such as cracks or coating delamination from a substrate. Thus, it is important to obtain a coating with low residual stress. Stress generation might have two natures:

(a) Thermal—during spraying, a high temperature is used, resulting in a change in phase composition and generating thermal stress in the main phase, which is used in WC coatings.
(b) Impact—in HVOF, a high speed of particles is achieved. When hot particles hit the substrate, additional stress is generated, which might have both linear and shear components.

Therefore, the generation of linear stress most likely has a thermal nature, related to thermal expansion of WC, while the generation of shear stress most likely has an impact nature. Such a phenomenon was not described earlier as an effect of relatively high hardness of used substrate materials (steel, cast iron, nickel alloys, etc.), resulting in cracking of WC particles during impact. Used magnesium alloy has the lowest Young’s modulus and hardness of all engineering alloys and might deform during the HVOF process.

**Figure 16:**
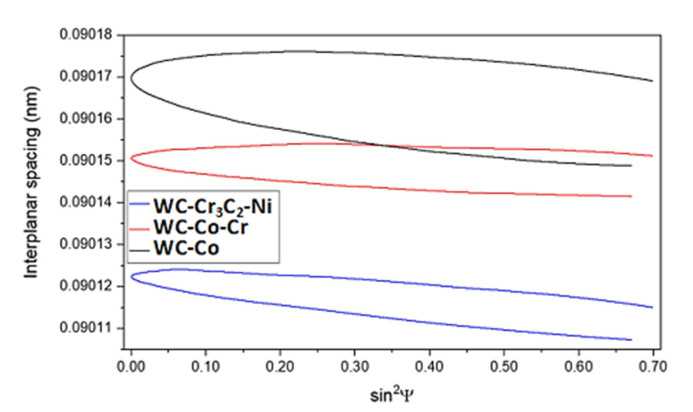
Residual stress diagrams were obtained for examined cermet coatings.

In all cermet coatings, linear stress shows a compressive nature (Figure 16, Table 3). In C2 and C3, residual stresses have a similar value, with a different part of shear stress contribution, while in C1, linear and shear stresses are almost even. However, shear stress contribution in cermet is very high, most likely as an effect of spraying onto magnesium substrate, which might deform during the HVOF process. In C2, shear stress is higher than in C1 (Figure 16), most likely due to the presence of chromium in C2 cermet, which might partially absorb energy during the HVOF process. The lowest shear stress contribution was detected in C3 coatings, most likely as an effect of the Cr_3_C_2_ carbide presence in powder, which may also absorb the impact energy (see Table 3).

Due to the complex nature of presence stress, it is nearly impossible to determine the order of the overall stress. However, compressive stress in HVOF coatings is unlikely to be eliminated; thus, only shear stress should be considered. Furthermore, it is a new aspect in HVOF-derived coatings. The C3 coating should be considered the best one because it is almost a shear stress-free material. On the other hand, in C2 coatings, high shear stress might have a negative impact on tribological properties, resulting in cracking of WC.

Observed results of residual stress (Figure 16) in the WC phase are in agreement with literature data describing residual stress in cermet coatings with similar thickness [24,25,45,46,47]. Oladijo et al. [25] observed residual stress of WC-Co coatings thermally sprayed onto different metal substrates, and residual stress was in the range of −130 MPa (an aluminum substrate) to −50 MPa (brass substrate). Książek et al. [46] calculated stress in a Cr_3_C_2_-NiCr coating in the range of −230 to −420 MPa, but Cr_3_C_2_ carbide has a much lower Young’s modulus than WC carbide. On the other hand, Masoumi et al. [45] detected residual stress around −130 MPa in 400 μm thick WC-Co-Cr coating, but they used E = 316 GPa in their calculations. Santana et al. [24] show that in WC-Co coatings with thickness in the range of 300–450 μm, residual stresses are −180 to −220 MPa.

## 4. Conclusions

This work was focused on HVOF spraying with feedstock powders WC-Co-Cr, WC-Co and WC-Cr_3_C_2_-Ni. The coatings were studied in terms of the influence of feedstock powder content on the microstructure, phase composition and residual stress.

It can be summarized that:

1. All the coatings revealed relatively smooth, dense and homogeneous structure. In all samples, the interface between the coating and magnesium alloy substrate was clear, and no evidence of delamination was observed.
2. The porosity in all of the investigated coatings was quite similar (in vol %)—2.9 ± 0.7 for C1, 2.6 ± 0.5 for C2 and 1.9 ± 0.5 for C3—and the thickness was in the range of 177 ± 20 µm to 279 ± 24 µm. In addition, the lowest microhardness (HV0.3) was observed for the C3 sample (989 ± 124), while the highest was observed for the C2 (1269 ± 167).
3. Based on the results of the TEM analysis, the C1 coating contains an amorphous matrix and two types of precipitates: WC and W_2_C. The C2 coating contains a matrix with an amorphous structure and precipitation of WC. Finally, analysis of the C3 coating showed a matrix with an amorphous structure and two types of precipitations: WC and Cr_3_C_2_.
4. XRD studies showed that phase composition of cermet coatings consists of hexagonal WC, hexagonal W_2_C carbide, hexagonal cobalt and a cubic solid solution of tungsten in cobalt with composition Co_0.9_W_0.1_. Additionally, in the WC-Cr_3_C_2_-Ni coating, Cr_3_C_2_ and Cr_7_C_3_ carbides were identified.
5. In all cermet coatings, linear stress shows a compressive nature. However, in C2 and C3, residual stress has a similar value, with a different part of shear stress contribution, while in C1, both linear and shear stresses are almost even and lower than in other coatings.

## Figures and Tables

**Table 1:** Spraying parameters of cermet coatings.

Oxygen Flow Rate, L/min	900
Kerosene flow rate, L/h	26.1
N_2_ flow rate, L/min	12
Powder feed rate, g/min	70
Water flow rate, L/min	23
Spray distance, mm	360

**Table 2:** Average thickness, porosity and microhardness of deposited coatings.

	C1	C2	C3
Thickness, µm	279 ± 24	206 ± 8	177 ± 20
Porosity, vol %	2.9 ± 0.7	2.6 ± 0.5	1.9 ± 0.5
HV0.3	1198 ± 195	1269 ± 167	989 ± 124

**Table 3:** Mean residual stress values in various cermet coatings sprayed onto AZ31 magnesium substrate.

Sample	Residual Stress, MPa	
	Linear Stress	Shear Stress
C1	−65.0 ± 28.8	56.5 ± 25.1
C2	−109.3 ± 29.3	86.1 ± 33.4
C3	−113.8 ± 3.7	27.6 ± 11.5

## Data Availability

Not applicable.
